# Clinical Challenges in Managing Shoulder Osteoarthritis and Suprascapular Neuropathy

**DOI:** 10.7759/cureus.99345

**Published:** 2025-12-16

**Authors:** José Eduardo Sousa, Nuno Madureira, Paula Freire, Matilde Rodrigues, Andreia Morgado

**Affiliations:** 1 Physical Medicine and Rehabilitation, Centro de Medicina de Reabilitação da Região Centro - Rovisco Pais, Coimbra, PRT

**Keywords:** muscle atrophy, shoulder arthritis, shoulder pain management, shoulder rehabilitation, suprascapular neuropathy

## Abstract

Chronic shoulder pain is a common and multifactorial condition. Suprascapular nerve neuropathy, although relatively uncommon, may lead to pain, weakness, and muscle atrophy as a result of extrinsic compression, trauma, or inflammation. When it occurs in the presence of glenohumeral osteoarthritis, overall shoulder function may be further compromised, complicating both diagnostic assessment and therapeutic decision-making.

We report the case of a 56-year-old man with a three-year history of chronic left shoulder pain and progressive proximal weakness, despite multiple courses of physiotherapy. His only relevant precedent trauma was a traumatic brain injury 20 years earlier. Physical examination revealed atrophy of the supraspinatus and infraspinatus muscles, reduced strength in lateral elevation and external rotation, and diffuse pain during shoulder maneuvers. Plain radiographs demonstrated glenohumeral osteoarthritis without evidence of significant rotator cuff arthropathy. Ultrasound imaging showed joint effusion, a paralabral cyst, and features of advanced osteoarthritis. Electroneuromyography revealed denervation of the supraspinatus and infraspinatus muscles, consistent with suprascapular neuropathy at the suprascapular notch. Glenohumeral arthrocentesis provided only transient symptomatic relief, and the patient was subsequently referred for orthopedic evaluation for potential reverse shoulder arthroplasty.

This case highlights the importance of recognizing the potential interplay between suprascapular neuropathy and glenohumeral osteoarthritis in patients presenting with chronic shoulder pain. It also emphasizes the diagnostic value of integrated imaging and electrophysiological assessment to guide appropriate management and optimize patient outcomes.

## Introduction

Chronic shoulder pain is one of the most frequent musculoskeletal complaints in clinical practice and may arise from various articular, tendinous, or neurological causes [1]. Suprascapular nerve neuropathy is rare but clinically significant, often presenting with pain, weakness, and muscle atrophy [2,3].

From an anatomical standpoint, the suprascapular nerve originates from the upper trunk of the brachial plexus (C5-C6), passing through two narrow fibro-osseous regions, the suprascapular notch and the spinoglenoid notch, where it is particularly vulnerable to compression. These anatomical constraints become clinically relevant in the presence of space-occupying lesions, repetitive mechanical stress, rotator cuff pathology, or inflammatory processes, which may compromise nerve conduction [2,3].

Multiple etiologies may precipitate nerve compression, including trauma, repetitive overhead loading, and degenerative or inflammatory changes at the shoulder girdle [1,2]. Paralabral cysts, commonly associated with superior or posterosuperior labral tears, may exert mass effect at the suprascapular or spinoglenoid notch, resulting in extrinsic nerve compression. Similarly, joint effusion caused by glenohumeral inflammation or degenerative arthropathy increases local pressure and further heightens the risk of neuropathy [4,5].

When suprascapular neuropathy coexists with glenohumeral osteoarthritis, functional impairment can be amplified due to the cumulative effects of biomechanical alteration, capsulolabral degeneration, and muscular imbalance. Osteoarthritic progression is frequently accompanied by osteophyte formation, synovitis, labral degeneration, and joint effusion, phenomena that may promote paralabral cyst formation and secondary nerve compression [4,5]. In such scenarios, clinical diagnosis becomes more challenging because symptoms of neuropathy may overlap with those originating from joint degeneration [3].

This case report describes a patient with concurrent glenohumeral arthropathy and suprascapular neuropathy, emphasizing the diagnostic complexity and therapeutic considerations associated with this clinical presentation.

## Case presentation

A 56-year-old man with a medical history of gout and a remote traumatic brain injury occurring 20 years earlier presented to the physical medicine and rehabilitation department with a three-year history of chronic left shoulder pain of mixed mechanical and inflammatory characteristics. The pain had a baseline intensity of 5/10 on the Visual Analog Scale and progressively became associated with proximal shoulder weakness. Despite adherence to conservative management, including analgesics and a structured physical therapy program consisting of glenohumeral and scapular mobility exercises, rotator cuff and scapular stabilizer strengthening, postural re-education, stretching, and pain modalities, he reported only minimal and short-lasting pain relief. Over time, the symptoms increasingly affected his activities of daily living, particularly tasks requiring overhead elevation or external rotation, such as dressing or combing his hair. He denied any trauma during this period or signs of systemic infection.

On physical examination, the patient exhibited marked wasting of the supraspinous and infraspinous fossae, with loss of posterior shoulder contour. Strength testing revealed Medical Research Council grade 3 weakness, predominantly affecting shoulder abduction and external rotation. Active and passive ranges of motion were both limited, particularly abduction (up to 100º) and external rotation (up to 10º). Palpation elicited diffuse tenderness around the glenohumeral region, and generalized pain was reproduced during rotator cuff testing. No signs of instability were detected.

**Figure 1:**
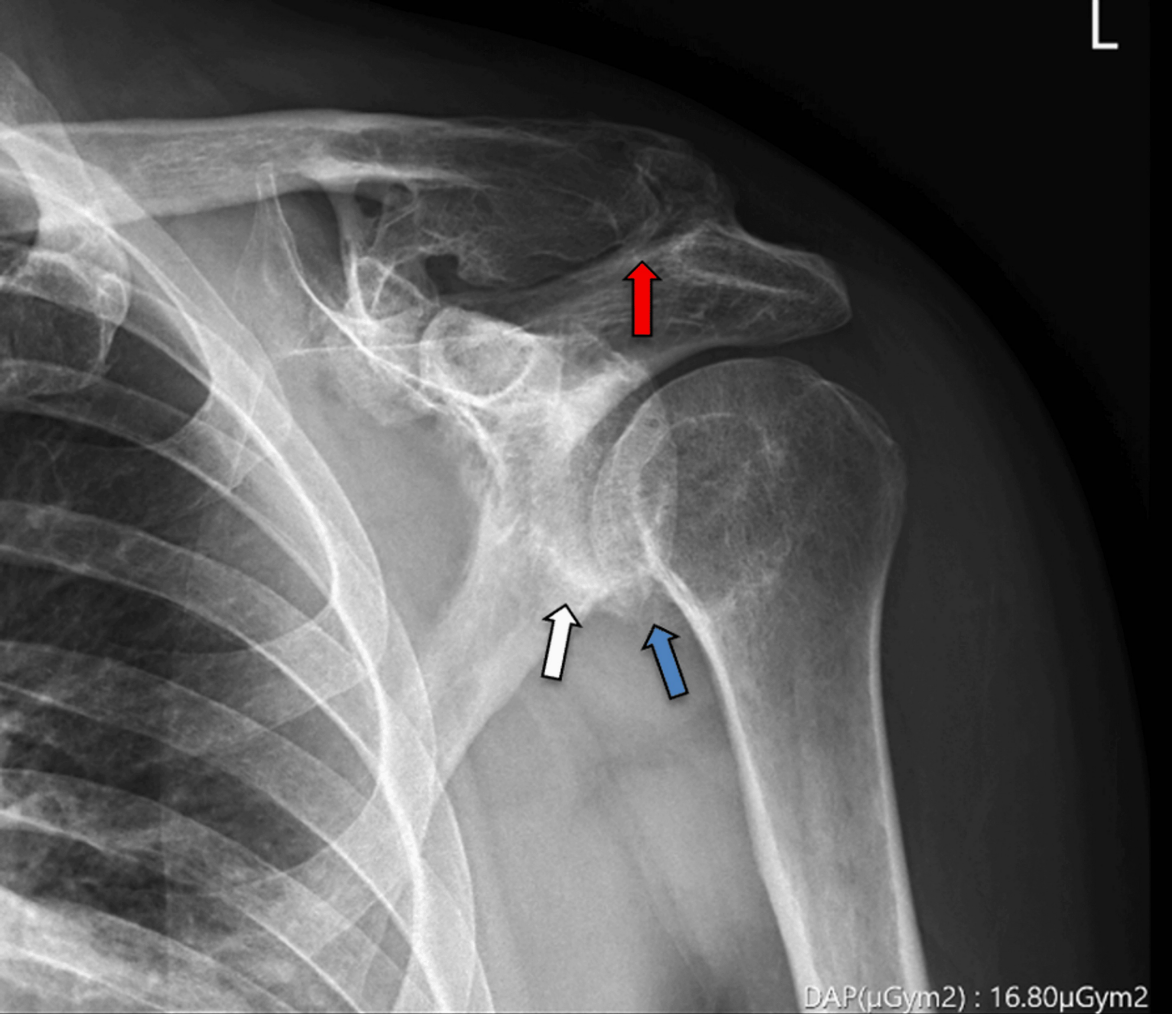
Standard anteroposterior shoulder radiograph Acromioclavicular (red arrow) and glenohumeral osteoarthritis findings with prominent osteophyte formation (blue arrow) and subchondral sclerosis (white arrow).

Standard radiographs revealed advanced acromioclavicular and glenohumeral osteoarthritis (Figure 1).

**Figure 2:**
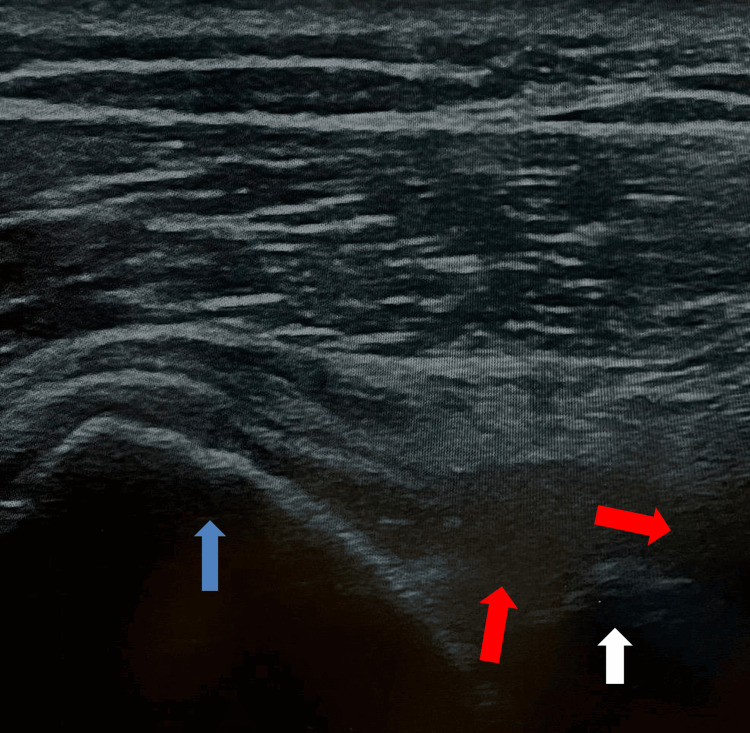
Ultrasound findings with joint effusion and a suspected paralabral cyst projecting into the spinoglenoid notch Blue arrow (humeral head); red arrows (joint effusion and paralabral cyst with extension to the spinoglenoid notch); white arrow (scapula).

Shoulder ultrasound demonstrated effusion within the long head of the biceps tendon, suggestive of intra-articular joint effusion; signs of tendinosis and partial rupture of the subscapularis and supraspinatus tendons with extensive subcoracoid and subacromial bursae; cortical bone irregularities; joint effusion; and a suspected paralabral cyst projecting into the spinoglenoid notch, consistent with potential extrinsic nerve compression at this point (Figure 2). Ultrasound did not accurately assess whether a shoulder effusion extended to the suprascapular notch.

**Figure 3:**
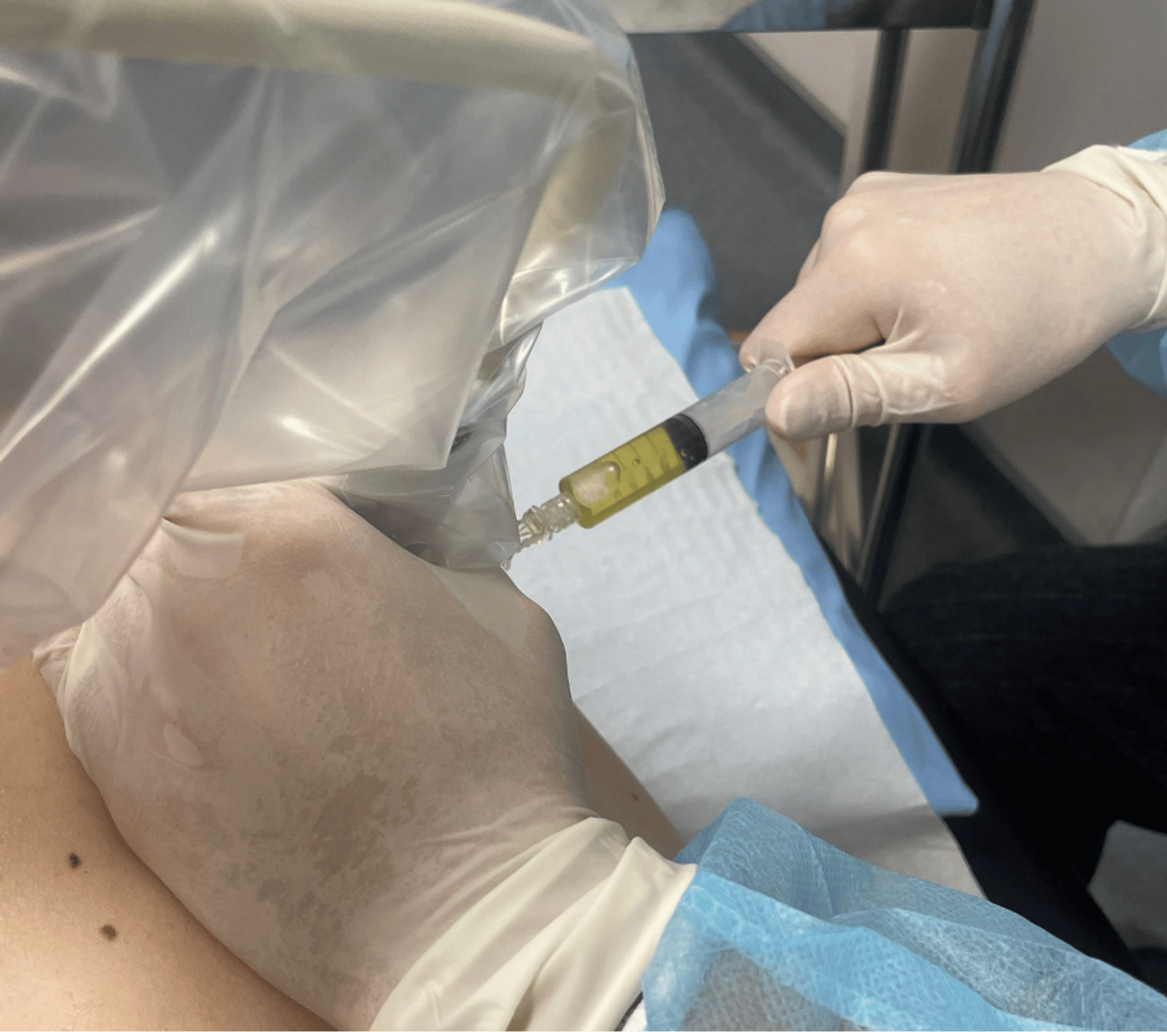
Artrhocentesis with viscous, yellow clear fluid aspiration

Diagnostic arthrocentesis yielded clear, viscous, sterile synovial fluid without crystals (Figure 3, Table 1), resulting in only transient partial pain relief.

**Table 1:** Arthrocentesis results Findings consistent with inflammatory (non-septic) synovitis of the shoulder joint.

Parameter	Result	Reference range/interpretation
Appearance	Slightly cloudy, yellow	Normal: clear, pale yellow
Viscosity	Increased	Normal: high (string test positive)
Total leukocyte count	24,800 cells/µL	Normal: <200 cells/µL - consistent with inflammatory synovitis
Differential count	Neutrophils: 68%, lymphocytes: 25%, monocytes/macrophages: 7%	Inflammatory pattern; neutrophil-predominant
Erythrocyte count	400 cells/µL	Mild increase - may reflect minor trauma or inflammation
Glucose (synovial/serum)	78 mg/dL/90 mg/dL	Mildly decreased - consistent with inflammation
Protein	4.3 g/dL	Elevated - consistent with synovial inflammation
Crystals (polarized light)	None detected	No evidence of crystal arthropathy
Gram Stain	No organisms seen	Negative for bacterial infection
Culture	No growth after 48 hours	Confirms non-infectious synovitis

Due to clinical and ultrasound findings, the history of polytrauma and severe muscle atrophy electromyography (EMG) was requested and revealed active denervation potentials in the supraspinatus and infraspinatus muscles, with reduced recruitment patterns, confirming suprascapular nerve involvement at the suprascapular notch (Table 2) likely secondary to extrinsic compression (e.g., joint effusion, paralabral cyst, or bursal extension) associated with glenohumeral osteoarthritic changes.

**Table 2:** EMG results Findings consistent with chronic, partial axonal suprascapular neuropathy, most likely at the suprascapular notch: active denervation and chronic reinnervation in both the supraspinatus and infraspinatus muscles. Preservation of deltoid, biceps, and trapezius function, excluding axillary, musculocutaneous, or spinal accessory nerve involvement, was confirmed, such as normal sensory studies, excluding brachial plexus or cervical root pathology (C5–C6). EMG: electromyography, MUPs: motor unit potentials

Muscle	Insertional activity	Spontaneous activity	Motor unit potentials	Recruitment pattern	Comment
Supraspinatus	Increased	+++ fibrillation potentials, positive sharp waves	Large-amplitude, long-duration polyphasic units	Reduced	Active denervation with chronic reinnervation
Infraspinatus	Increased	++ fibrillation potentials	Large, polyphasic MUPs	Reduced	Active denervation with reinnervation
Deltoid	Normal	None	Normal	Normal	Intact axillary nerve
Biceps brachii	Normal	None	Normal	Normal	Intact musculocutaneous nerve
Trapezius	Normal	None	Normal	Normal	Intact spinal accessory nerve
Cervical paraspinals	Normal	None	Normal	Normal	No evidence of radiculopathy

The overall diagnostic workup strongly indicated that the coexistence of advanced glenohumeral osteoarthritis and secondary suprascapular neuropathy best explained the patient’s symptoms. Imaging demonstrated significant degenerative joint changes, together with extrinsic compression of the suprascapular nerve at the suprascapular notch, potentially caused by a space-occupying paralabral cyst and associated joint effusion. This combination of structural degeneration and nerve entrapment provided a coherent explanation for the patient’s progressive pain, weakness, and functional decline.

Given the severity of joint degeneration and the resulting functional impairment, the patient was referred to the orthopedic department to assess suitability for reverse shoulder arthroplasty. Integrity of the axillary nerve was confirmed by EMG, an essential consideration because postoperative functional recovery in this procedure relies primarily on deltoid strength. As reverse shoulder arthroplasty is the treatment of choice in patients with shoulder osteoarthritis with rotator cuff insufficiency, this confirmation supported surgical planning and the anticipated rehabilitation trajectory.

## Discussion

Suprascapular neuropathy should be suspected in patients presenting with shoulder weakness, muscle atrophy, and poorly localized pain, particularly when imaging reveals degenerative changes such as joint effusion or paralabral cysts near the suprascapular or spinoglenoid notch [2,3]. EMG and nerve conduction studies are essential to confirm neuropathy and localize the lesion, while imaging (ultrasound or MRI) helps identify underlying structural causes [2,3].

The clinical presentation of suprascapular neuropathy can closely mimic several other shoulder and cervical disorders, making a thorough differential diagnosis essential for appropriate management [1,4,5]. Because pain, weakness in abduction and external rotation, and muscle atrophy are common to multiple conditions, careful clinical evaluation supported by electrodiagnostic and imaging studies is crucial [2,3].

Differential diagnoses include cervical radiculopathy, particularly involving the C5-C6 roots, brachial plexus lesions affecting the upper trunk or posterior cord, and complete rotator cuff tears, all of which can cause overlapping mechanical weakness and atrophy but were excluded based on our complementary diagnostic workup [5]. Axillary nerve neuropathy should also be carefully assessed, especially in post-traumatic or postsurgical contexts, and critically when planning for reverse shoulder arthroplasty [4,5]. This is because the success of reverse shoulder arthroplasty in patients with rotator cuff insufficiency and glenohumeral osteoarthritis depends primarily on the function of the deltoid muscle. As the axillary nerve innervates the deltoid, its integrity is essential to ensure adequate postoperative shoulder elevation and functional recovery [4,5].

Management of suprascapular neuropathy depends on symptom severity and the underlying etiology. Treatment options may include physiotherapy, pain management, which proved ineffective in this case, or nerve decompression; however, in the presence of advanced glenohumeral osteoarthritis with active and passive range of motion limitations combined with rotator cuff deficiency, surgical intervention, specifically reverse shoulder arthroplasty, was preferred over isolated suprascapular nerve decompression [4,5].

## Conclusions

This case highlights the complexity of chronic shoulder pain when neuromuscular deficits coexist with degenerative joint changes. The simultaneous presence of glenohumeral arthropathy and suprascapular neuropathy presents significant diagnostic and therapeutic challenges, as overlapping symptoms can obscure the primary etiology.

A thorough clinical assessment, supported by targeted complementary studies, such as EMG to confirm and localize neuropathy, and imaging modalities, including ultrasound, radiography, or MRI, to identify structural abnormalities, is essential to delineate potential sites of nerve entrapment and to evaluate the extent of degenerative involvement. Recognizing suprascapular neuropathy in this context allows for appropriate and timely intervention, guiding surgical or conservative management and optimizing functional outcomes.
